# Vulval elephantiasis as a result of tubercular lymphadenitis: two case reports and a review of the literature

**DOI:** 10.1186/1752-1947-4-369

**Published:** 2010-11-18

**Authors:** Chintamani, JP Singh, Megha Tandon, Rohan Khandelwal, Tushar Aeron, Sidharth Jain, Nikhil Narayan, Rahul Bamal, Yashwant Kumar, S Srinivas, Sunita Saxena

**Affiliations:** 1 Department of Surgery, Vardhman Mahavir Medical College, Safdarjang Hospital, New Delhi, India; 2 Institute of Pathology, Indian Council of Medical Research, Vardhman Mahavir Medical College, Safdarjang Hospital, New Delhi, India

## Abstract

**Introduction:**

Elephantiasis as a result of chronic lymphedema is characterized by gross enlargement of the arms, legs or genitalia, and occurs due to a variety of obstructive diseases of the lymphatic system. Genital elephantiasis usually follows common filariasis and lymphogranuloma venereum. It may follow granuloma inguinale, carcinomas, lymph node dissection or irradiation and tuberculosis but this happens rarely. Vulval elephantiasis as a consequence of extensive lymph node destruction by tuberculosis is very rare. We present two very unusual cases of vulval elephantiasis due to tuberculous destruction of the inguinal lymph nodes.

**Case presentation:**

Two Indian women - one aged 40 years and the other aged 27 years, with progressively increasing vulval swellings over a period of five and four years respectively - presented to our hospital. In both cases, there was a significant history on presentation. Both women had previously taken a complete course of anti-tubercular treatment for generalized lymphadenopathy. The vulval swellings were extremely large: in the first case report, measuring 35 × 25 cm on the right side and 45 × 30 cm on the left side, weighing 20 lb and 16 lb respectively. Both cases were managed by surgical excision with reconstruction and the outcome was positive. Satisfactory results have been maintained during a follow-up period of six years in both cases.

**Conclusions:**

Elephantiasis of the female genitalia is unusual and it has rarely been reported following tuberculosis. We report two cases of vulval elephantiasis as a consequence of extensive lymph node destruction by tuberculosis, in order to highlight this very rare clinical scenario.

## Introduction

Elephantiasis, the result of chronic lymph edema, is characterized by gross enlargement of the arms, legs or genitalia, and it occurs due to a variety of obstructive diseases of the lymphatic system. Genital elephantiasis is a common result of filariasis and lymphogranuloma venereum. However, it may also follow granuloma inguinale, carcinomas, lymph node dissection or irradiation and tuberculosis, although this happens rarely [1-6]. Filarial elephantiasis of the female genitalia is extremely uncommon; a rough estimate of its incidence would be no more than one to two percent of the total cases of filarial elephantiasis [7]. Elephantiasis of the female genitalia due to other causes is rarer still. We present two unusual cases of vulval elephantiasis as a consequence of extensive lymph node destruction by tuberculosis.

## Case presentation

### Case report 1

A 40-year-old Indian woman presented with progressively increasing vulval swellings over a period of five years. She also described a loss of appetite and weight. There was a history of fever with a rise in the evenings, night sweats and vaginal discharge, although her menstrual periods were normal. Eight years prior to presentation, she had generalized lymph node tuberculosis with discharging cervical and inguinal sinuses, for which she received a full course of anti-tubercular therapy. Her tuberculosis was completely cured by the anti-tubercular therapy and she did not show any evidence of a recurrence. Her genital swellings were extremely large and caused her to experience difficulty in walking. Sexual intercourse was not possible.

**Figure 1:**
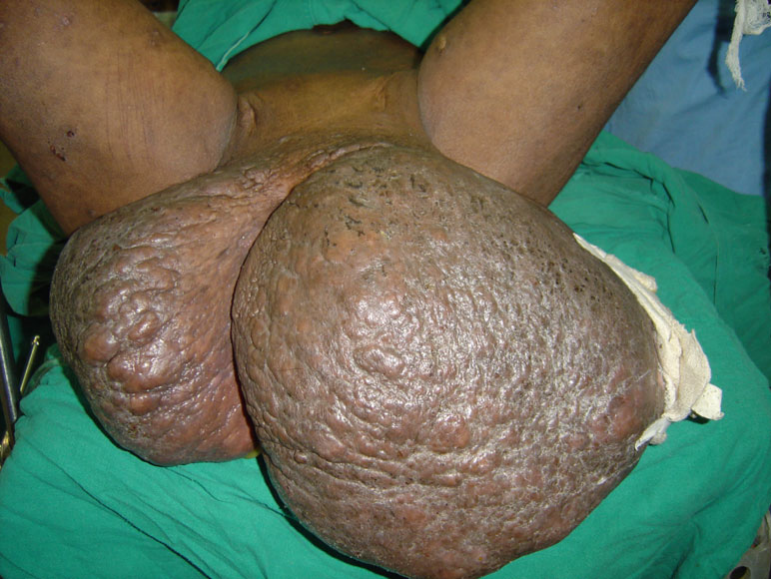
**Vulval elephantiasis involving both labia majora (35 × 25 cm on the right side and 45 × 30 cm on the left side) with well-healed scars of previously discharging inguinal sinuses**. The lower limbs are normal (Case report 1).

**Figure 2:**
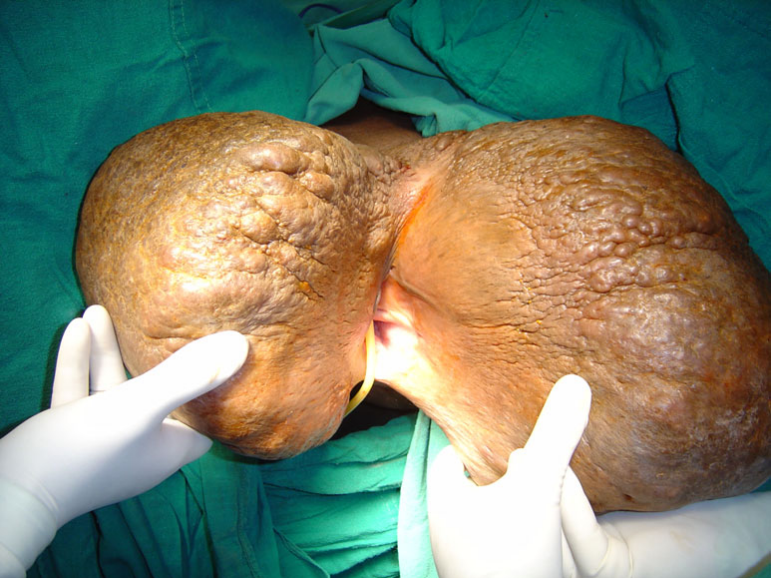
**The pre-operative appearance (Case report 1)**.

Our examination revealed that she was poorly nourished, with a marked pallor. There was no lower limb edema. She had two giant vulval swellings measuring 35 × 25 cm on the right side and 45 × 30 cm on the left side (Figure 1 and 2). In the standing position, the left vulval swelling extended below her knees. While walking, she had to tuck the swellings between her buttocks. The skin overlying the swellings was thick and rugose.

Both her inguinal and cervical regions had puckered scars of healed sinuses without any palpable lymph node. The rest of our physical examination, including her vaginal wall, chest and abdomen was normal.

She was found to be severely anemic (Hb 6 gm%) with a normal leucocyte count. Our other investigations, including blood urea nitrogen, serum electrolytes, creatinine, Mantoux test, night blood smear, chest X-ray, ultrasonography of her abdomen and pelvis, and pap smear were normal.

**Figure 3:**
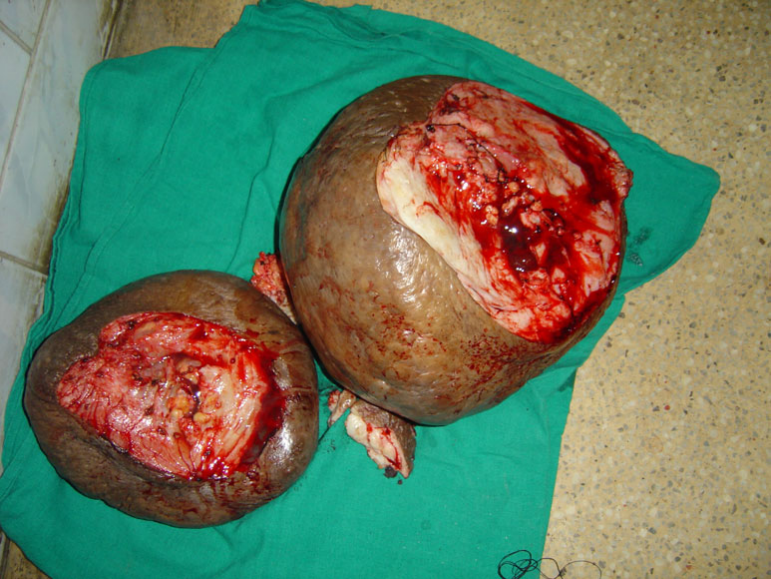
**The resected specimen (the left weighing 20 pounds and the right weighing 16 pounds) (Case report 1)**.

**Figure 4:**
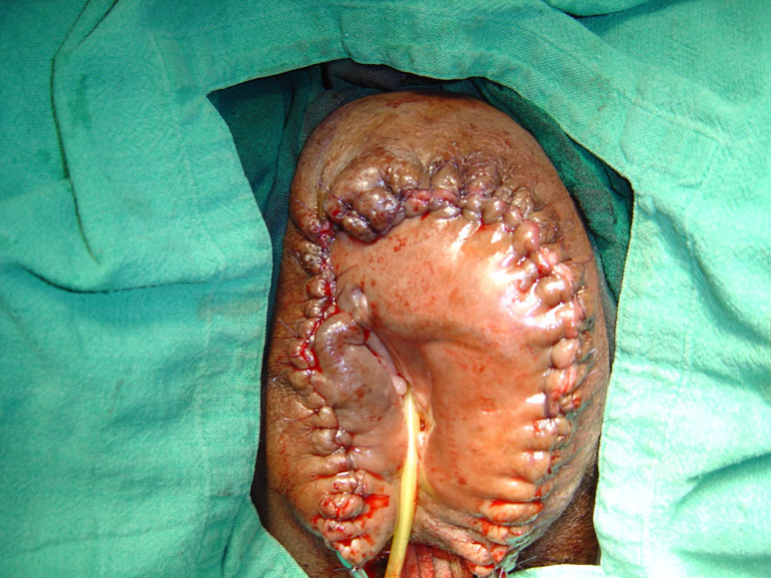
**Post-operative appearance after resection of the lesions (Case report 1)**.

**Figure 5:**
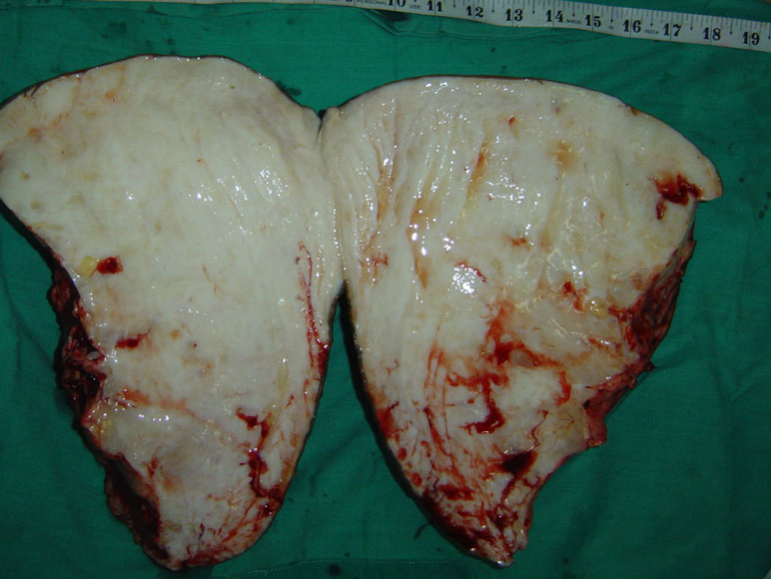
**The specimen bisected, showing grayish-white tissue loaded with lymph (Case report 1)**.

She was taken up for surgery after the correction of her anemia. A wide local excision with a primary closure was performed. Part of the fibro-fatty tissue of her labia majora was preserved to give them a natural bulging appearance (Figures 2, 3, 4, 5). There was considerable oozing of lymph during surgery and in the post-operative period, but healing occurred with primary intention. The swellings removed from her right and left labia weighed 20 lb and 16 lb respectively. The immediate post-operative period was uneventful. Nearly six years of follow up revealed a satisfactory recovery, although in the immediate post-operative period and in the early follow-up period she presented with seroma formation under the skin flaps that was managed by aspiration and pressure bandaging. She also experienced episodes of serous discharge from the site that was self limiting and was managed by pressure bandaging.

### Case report 2

A 27-year-old Indian woman presented to our hospital with a very similar history and findings to that of case report 1, although her swellings were not as large. She presented with progressively increasing vulval swellings over a period of four years. She had a past history of fever, night sweats, weight and appetite loss, and vaginal discharge. Her menses were normal. Seven years prior to presentation, she also had generalized lymph node tuberculosis with discharging cervical and inguinal sinuses, for which she had received a full course of anti-tubercular therapy. Her tuberculosis was completely cured by the anti-tubercular therapy and she did not show any evidence of a recurrence.

**Figure 6:**
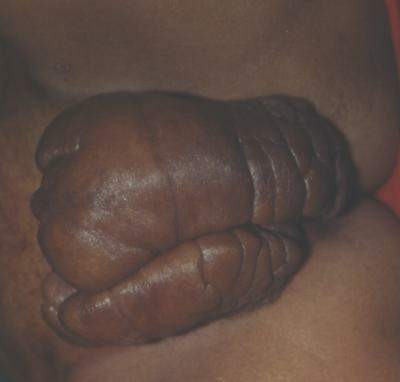
**Vulval elephantiasis involving both of the labia majora (15 × 7 cm labial swelling on the left side and 9 × 5 cm labial swelling on the right side)**. The lower limbs are normal (Case report 2).

**Figure 7:**
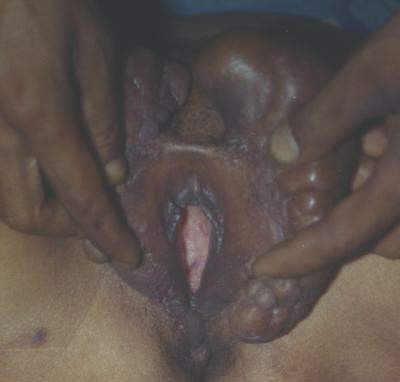
**A closer view of the labia (Case report 2)**.

**Figure 8:**
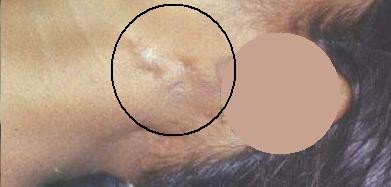
**Well-healed scars of old cervical tubercular sinuses (Case report 2)**.

Her general physical examination was normal and there was no lower limb edema. She had a 15 × 7 cm labial swelling on the left side and a 9 × 5 cm labial swelling on the right side (Figure 6). The skin overlying the swellings was thickened. She had the puckered scars of healed sinuses without any palpable lymph node in the inguinal and cervical regions (Figures 7 and 8). The rest of the physical examination, including her vaginal wall, chest and abdomen, was normal.

All of our routine investigations, including hemoglobin, total and differential cell counts, blood urea nitrogen, Mantoux test, night blood smear, chest X-ray, ultrasound of her abdomen and pelvis, and pap smear were normal.

She was taken up for surgery and a wide local excision with primary closure was performed. Both of her labia majora were given a natural soft and bulging appearance. There was slight oozing in the post-operative period, but healing occurred with primary intention (i.e.the incisions that were closed with sutures healed normally). Her post-operative period was uneventful. A follow-up period of six years revealed a satisfactory recovery and, unlike case report 1, she experienced minimal discharge from the wound site and her recovery was uneventful.

A histopathological examination of the specimen in both cases showed changes of lymphedema. The features were suggestive of non-specific inflammation. There was, however, no clear evidence of tuberculosis in the specimens (in the form of granulomas and/or acid fast bacilli), malignancy, filariasis or donovanosis(Figure 5).

## Discussion

Lymphedema occurs due to an inability of the existing lymphatic system to accommodate the protein and fluid entering the interstitial compartment at tissue level. In the first stage of lymphedema, impaired lymphatic drainage results in protein-rich fluid accumulation in the interstitial compartment. Clinically, this manifests as soft-pitting edema. In the second stage, there is an accumulation of fibroblasts, adipocytes and macrophages in the affected tissues, culminating in a local inflammatory response. This results in a deposition of the connective tissue and adipose elements at the skin and subcutaneous level, leading to non-pitting edema. In the third and most advanced stage, the affected tissues sustain further injury as a result of both the local inflammatory response and recurrent infections. Such repeated episodes injure the remaining, incompetent lymphatic channels, progressively worsening the underlying insufficiency of the lymphatic system. This eventually results in excessive subcutaneous fibrosis and scarring with associated severe skin changes characteristic of lymphostatic elephantiasis [9,10].

Lymphedema is generally classified as primary when there is no known etiology, and as secondary when its cause is a known disease [9]. Primary lymphedema with onset before two years of age is referred to as congenital; the familial version of which is known as Milroy's disease. Primary lymphedema with onset between two and 35 years of age is called lymphedema praecox. It is the most common form of primary lymphedema, accounting for 80 percent of the cases. The familial version of lymphedema praecox is known as Meige's disease. Primary lymphedema with onset after 35 years of age is called lymphedema tarda. In general, primary lymphedema progresses more slowly than secondary lymphedema [9,10].

The most common form of lymphedema is secondary lymphedema. In developed countries, the most common causes of secondary lymphedema involve resection or ablation of the regional lymph nodes by surgery, radiation, tumor invasion, direct trauma, or, less commonly, an infectious process. Globally, filariasis, caused by infestation of the lymph nodes by the parasite *Wuchereria bancrofti*, is the most common cause of secondary lymphedema [10,11].

Vulval tuberculosis leading to pseudoelephantiasis - direct infiltration of the vulva by tuberculosis - is rare; however a few cases have been previously reported [10]. Vulval elephantiasis as a consequence of extensive lymph node destruction by tuberculosis in the inguinal region is rarer still. Sharma *et al*. reported two cases of vulval elephantiasis as a consequence of tubercular lymphadenitis, however, both the cases had smaller-sized vulval swellings [6]. In our case reports, the absence of a tubercular histology from the vulva rules out direct infiltration; that is, pseudoelephantiasis. Moreover, both of our cases had a past history of lymph node tuberculosis, with evidence of the puckered scars of healed sinuses in their inguinal regions. The etiology in both of our cases was the extensive destruction of the inguinal lymph nodes and their channels as a result of past tuberculosis, leading to a blockage of lymphatic drainage and resulting in vulval elephantiasis.

## Conclusions

Vulval elephantiasis is very rare, and vulval elephantiasis as a consequence of lymph node destruction by tuberculosis, as evidenced in our case reports, is rarer still. We present our cases to draw attention to this rare condition.

## Competing interests

The authors declare that they have no competing interests.

## Authors' contributions

C was the chief operating surgeon who analyzed and interpreted the patient data. JPS, MT, RK, TA, SJ, NN, RB, YK and SSr were the surgical residents who assisted in the surgery and work up of both of the patients. They also contributed to the preparation of the manuscript. SSa was the histopathologist who reported on the specimen. All authors read and approved the final manuscript.

## Consent

Written informed consent was obtained from the patients for publication of these case reports and any accompanying images. Copies of the written consents are available for review by the Editor-in-Chief of this journal.
